# Report of two protocol planned interim analyses in a randomised multicentre phase III study comparing capecitabine with fluorouracil and oxaliplatin with cisplatin in patients with advanced oesophagogastric cancer receiving ECF

**DOI:** 10.1038/sj.bjc.6602572

**Published:** 2005-05-31

**Authors:** K Sumpter, C Harper-Wynne, D Cunningham, S Rao, N Tebbutt, A R Norman, C Ward, T Iveson, M Nicolson, T Hickish, M Hill, J Oates

**Affiliations:** 1 Royal Marsden NHS Trust, Down's Road, Sutton, Surrey, UK; 2 Royal South Hants Hospital, Southampton, and Salisbury District Hospital, UK; 3 Oncology – Anchor Unit, Aberdeen Royal Infirmary, Aberdeen, UK; 4 Dorset Cancer Network, Royal Bournemouth Hospital, Bournemouth, UK; 5 Kent Oncology Centre, Hermitage Lane, Maidstone, UK

## Abstract

The purpose of the study was to establish the optimal dose of capecitabine (X) to be used within a multicentre, randomised study evaluating the potential roles of oxaliplatin (O) and X in chemonaive patients (pts) with advanced oesophagogastric cancer. Two by two design was used, and pts were randomised to one of four regimens and stratified for extent of disease, performance status (PS) and centre. The treatment regimens are epirubicin, cisplatin, 5-fluorouracil (ECF), EOF, ECX or EOX. Doses: E 50 mg m^−2^, C 60 mg m^−2^ and O 130 mg m^−2^ i.v. 3 weekly; F 200 mg m^−2^ day^−1^ i.v. and X 500 mg m^−2^ b.i.d.^−1^ (escalated to 625 mg m^−2^ b.i.d.^−1^ after results of first interim analysis) p.o., continuously. First interim analysis was performed when 80 pts had been randomised. Dose-limiting fluoropyrimidine toxicities were stomatitis, palmar plantar erythema (PPE) and diarrhoea; 5.1% of X-treated pts experienced grade 3/4 toxicity. Protocol planned dose escalation of X to 625 mg m^−2^ b.i.d.^−1^ was instituted and a second interim analysis has been performed; results are presented in this paper. A total of 204 pts were randomised at the time of the protocol planned 2nd interim analysis. Grade 3/4 fluoropyrimidine-related toxicity was seen in 13.7% pts receiving F, 8.4% pts receiving X 500 mg m^−2^ b.i.d.^−1^ and 14.7% pts receiving X 625 mg m^−2^ b.i.d.^−1^. Combined complete and partial response rates were ECF 31% (95% CI 18.7–46.3), EOF 39% (95% CI 25.9–53.1), ECX 35% (95% CI 21.4–50.3), EOX 48% (95% CI 33.3–62.8). Grade 3/4 fluoropyrimidine toxicity affected 14.7% of pts treated with X 625 mg m^−2^ b.i.d.^−1^, which is similar to that observed with F, confirming this to be the optimal dose. The replacement of C by O and F by X does not appear to impair efficacy. The trial continues to total accrual of 1000 pts.

Oesophagogastric (OG) cancer is the second most common cancer worldwide and while the incidence of gastric cancer is falling, that of adenocarcinoma of the OG junction is rising. The majority of pts present with inoperable or metastatic disease and consequently 5-year survival rates are only 10–15% (CRC, 1995). In advanced disease, palliative chemotherapy has been shown to improve survival and quality of life (QOL) when compared to best-supportive care alone (Murad *et al*, 1993; Pyrhonen *et al*, 1995; Glimelius *et al*, 1997).

One of the reference regimens for this disease is epirubicin, cisplatin and protracted venous infusion 5-fluorouracil (ECF) (Cunningham *et al*, 1991). This regimen was developed at the Royal Marsden Hospital, and in phase II studies response rates of 55–67% were observed (Evans *et al*, 1992; Harper *et al*, 1992; Melcher and Maughan, 1994; Zaniboni *et al*, 1995; Bamias *et al*, 1996). ECF, when compared to FAMTX in a prospective randomised clinical trial (RCT), demonstrated superior response rates (45 *vs* 21%), overall survival and QOL (Webb *et al*, 1997). A further RCT comparing ECF to MCF (substituting E with mitomycin C) confirmed similar efficacy for the two regimens: response rates ECF 42.2% and MCF 44.1% (*P*=0.692) and median survival 9.4 and 8.7 months, respectively (*P*=0.315). Global QOL, however, was superior with ECF (Ross *et al*, 2002).

Oxaliplatin (O) is a third-generation diaminocyclohexane platinum compound, proven to be active in various tumour types. Oxaliplatin has demonstrated synergy with 5-fluorouracil (5FU) in advanced colorectal cancer (Raymond *et al*, 2001). In the first-line treatment of advanced colorectal cancer, the combination of O and 5FU has response rates of 36–58% (Levi *et al*, 1992, 1994; De Gramont *et al*, 2000) and this is now an established treatment in this setting (Goldberg *et al*, 2004). In addition, activity of this combination has been demonstrated in advanced gastric cancer, with a response rate of 45% and a median overall survival of 8.6 months (Louvet *et al*, 2002). Also, in 26 pts previously treated with 5FU, with a bimonthly O and 5FU/LV bolus and infusional regimen, the response rate was 26% and the median overall survival was 7.3 months (Kim *et al*, 2003). Cisplatin is associated with peripheral neuropathy, renal toxicity, high frequency nerve deafness and emesis; it also requires intravenous hydration. Oxaliplatin does not cause any clinically significant renal toxicity or ototoxicity and the dose-limiting toxicity is a peripheral sensory neuropathy, which is usually reversible but cumulative. The unique activity of O in colorectal cancer, the preliminary interesting results in gastric cancer and its improved toxicity profile compared to cisplatin suggest the potential for improved results in OG cancer.

Capecitabine (X) is an oral fluoropyrimidine, which is absorbed from the gastrointestinal tract as an intact molecule, metabolised primarily in the liver and converted in tumour tissues to 5FU by the enzyme thymidine phosphorylase (found in higher concentrations in tumour cells than normal cells). In advanced colorectal cancer, two RCTs have shown X to be at least as effective as 5FU/LV (Mayo regimen) in terms of overall survival, with a better toxicity profile (Hoff *et al*, 2001; Van Cutsem *et al*, 2001). Capecitabine is known to be active in advanced gastric cancer, with a single agent response rate in chemonaive pts of 24% (Koizumi *et al*, 2003). A phase II study involving X 1250 mg m^−2^ day^−1^ D1–14 and cisplatin 60 mg m^−2^ day^−1^ D1 every 3 weeks, for first-line treatment of advanced gastric cancer, resulted in an overall response rate of 54% and a median survival of 10.1 months (Kim *et al*, 2002).

To achieve the synergistic effects of fluoropyrimidines in combination with platinum compounds, thymidylate synthase inhibition is required prior to platinum administration (Cho *et al*, 2002). Preclinical studies have confirmed that a twice daily dosing of X will ensure efficient thymidylate synthase inhibition. A continuous twice daily X monotherapy schedule has been studied in a phase I trial (Budman *et al*, 1998). The maximum tolerated dose was 1657 mg m^−2^ day^−1^ and the recommended dose for phase II studies was 1331 mg m^−2^ day^−1^. The conclusions of a phase I study of the combination of O and X in advanced solid tumours recommended a dose of X 1000 mg m^−2^ b.i.d.^−1^ D1–14 (80% of the monotherapy dose) in combination with O 130 mg m^−2^ every 3 weeks (Diaz-Rubio *et al*, 2002). The dose-limiting toxicity of the combination was diarrhoea and the combination resulted in limited additional haematological toxicity.

In this study, it was not expected that the proposed starting dose of X of 500 mg m^−2^ b.i.d.^−1^ continuously (75% of monotherapy dose) would lead to more toxicity than PVI 5FU 200 mg m^−2^. The purpose of this study was, therefore, to establish the optimal dose of X in the initial phase of this randomised trial by incorporating a dose escalation/de-escalation. We report the results of this initial phase and the subsequent planned second interim analysis.

## PATIENTS AND METHODS

### Study design

**Figure 1:**
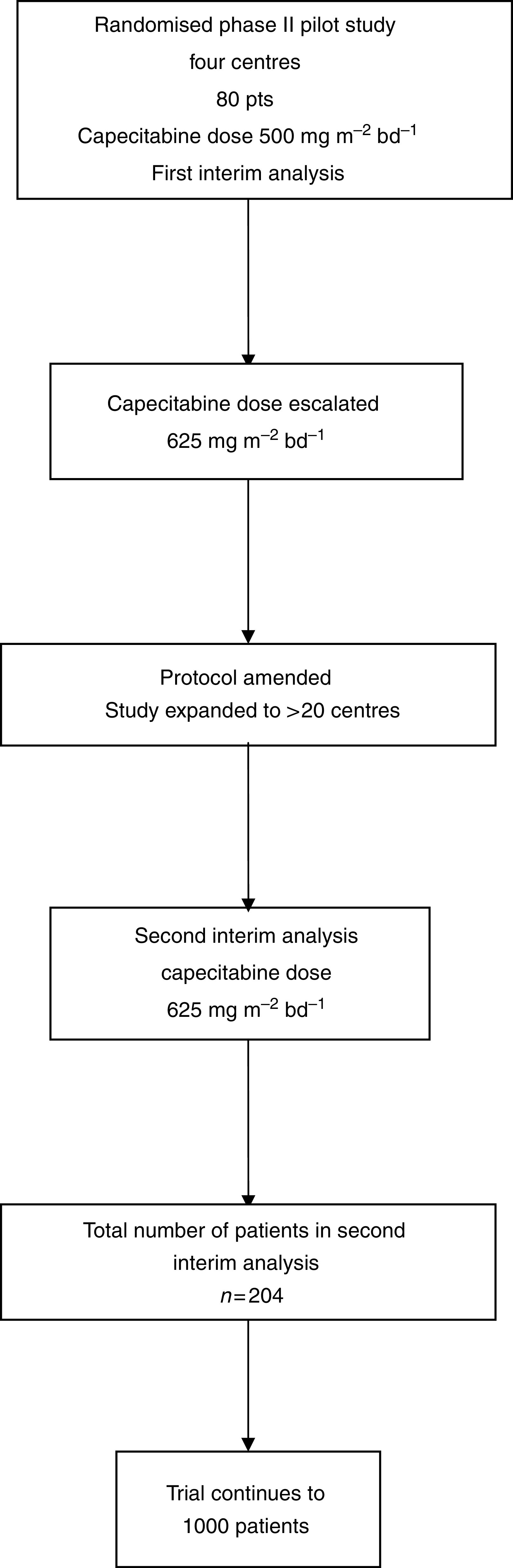
Trial design.

The study uses a two by two randomisation to compare X with PVI 5FU and O with cisplatin. The first 80 pts were randomised in a pilot phase II study, permitting dose escalation/de-escalation in the event of protocol-defined differences in fluoropyrimidine-dependent Common Toxicity Criteria (CTC) grade 3/4 toxicity in the X study arms. Fluoropyrimidine-related toxicity (stomatitis, palmar-plantar erythema (PPE) and diarrhoea) was analysed in a first planned interim analysis, according to fluoropyrimidine containing regimen. It was planned that if the grade 3/4 fluoropyrimidine-related toxicity of this dose of X was <10%, the dose of X would be increased by 25%, if the grade 3/4 fluoropyrimidine toxicity was 11–29%, the dose would remain unchanged and if it was >30% the dose would be reduced by 25%. Following determination of the dose of X to be used in the chemotherapy combinations (first interim analysis), the multicentre phase III trial was opened to recruitment and a second interim analysis was planned at 200 pts. This paper reports the results of the first and second preplanned interim analyses. Figure 1 summarises the trial design employed.

### Patient eligibility

Patients were required to have histologically verified locally advanced or metastatic adenocarcinoma, squamous cell or undifferentiated carcinoma of the oespohagus, oesophagogastric junction (OGJ) or stomach. The primary tumour was classified as inoperable on the basis of either findings at laparotomy or computed tomography (CT) scan and endoscopic ultrasound results. Patients could not have received any previous chemotherapy or radiotherapy unless the latter was adjuvant treatment with relapse outside the radiotherapy field. Patients were required to have adequate bone marrow (platelets>100 × 10^9^ l^−1^, WBC count>3 × 10^9^ l^−1^), renal (glomerular filtration rate ⩾60 ml min^−1^ and serum creatinine within normal range) and hepatic (bilirubin <2 × upper limit of normal range) function, Eastern Cooperative Oncology Group performance status 0–2, life expectancy of at least 3 months and no concurrent uncontrolled medical illness. If there was a suspicion of left ventricular dysfunction, amultigated cardiac scan was performed and pts were excluded if this was below the reference range for the institution. The study was approved by the Scientific and Research Ethics Committees of the participating institutions and all participants gave written informed consent before entering the study.

### Pretreatment evaluation

A full history was taken and an examination was performed on pts prior to treatment. Baseline full blood count, clotting screen, urea and electrolytes, liver function tests and carcinoembryonic antigen were performed in all pts. All pts had a baseline CXR and CT scan of chest, abdomen and pelvis within 28 days of commencing treatment. Upper GI endoscopy was performed at baseline unless pts had a histological diagnosis obtained at a laparotomy; baseline EDTA clearance or 24 h urinary creatinine clearance was measured prior to randomisation and baseline QOL was measured using the European Organisation for Research and Treatment of Cancer (EORTC) core 30 questionnaire.

### Study protocol

Dual lumen Hickman lines were inserted via the Seldinger technique, under local anaesthetic, in patient's randomised to either of the PVI 5FU containing combinations. On the day of the insertion of the Hickman line, pts were started on warfarin 1 mg day^−1^ as prophylaxis for line-related thrombosis. The four arms of treatment were as follows:

*ECF regimen*: Epirubicin 50 mg m^−2^ i.v. bolus every 3 weeks, cisplatin 60 mg m^−2^ with standard hydration every 3 weeks (Findlay *et al*, 1994), 5FU 200 mg m^−2^ daily by continuous infusion via central line.

*EOF regimen*: Epirubicin 50 mg m^−2^ i.v. bolus every 3 weeks, O 130 mg m^−2^ i.v. infusion over 2 h every 3 weeks, 5FU 200 mg m^−2^ daily by continuous infusion via central line.

*ECX regimen*: Epirubicin 50 mg m^−2^ i.v. bolus every 3 weeks, cisplatin 60 mg m^−2^ with standard hydration every 3 weeks (Findlay *et al*, 1994), X 500–625 mg m^−2^ b.i.d.^−1^ orally continuously.

*EOX regimen*: Epirubicin 50 mg m^−2^ i.v. bolus every 3 weeks, O 130 mg m^−2^ i.v. infusion over 2 h every 3 weeks, X 500–625 mg m^−2^ b.i.d.^−1^ orally continuously.

Antiemetics were routinely administered and it was recommended to participating centres that a 5HT3 antagonist and dexamethasone 8 mg i.v. be given prechemotherapy and dexamethasone 4 mg p.o. tds for 2 days and metoclopramide 10 mg tds for 3 days postchemotherapy. Planned treatment duration was 24 weeks. Response was evaluated at 12 and 24 weeks.

### Evaluation of toxicity

Toxicity was graded according to the National Cancer Institute common toxicity criteria (CTC) version 2. Dose modifications for ECF were made according to previously published guidelines (Findlay *et al*, 1994). Dose modifications of the E, cisplatin and PVI 5-FU in the EOF, ECX and EOX regimens were made according to the guidelines for ECF. Oxaliplatin was delayed for 1 week if neutrophil count <1.0 × 10^9^ l^−1^, platelet count <75 × 10^9^ l^−1^ or the patient had persistent grade 1 or 2 neuropathy. After recovery from grade 2–4 thrombocytopenia or grade 3/4 neutropenia, the dose of O was reduced to 100 mg m^−2^. On recovery of persistent grade 1/2 neuropathy between cycles or grade 3/4 neuropathy for 7–14 days, the dose of O was dose reduced to 100 mg m^−2^. In the event of persistent grade 3/4 neuropathy, further O was omitted and carboplatin could be substituted at the investigators discretion. If laryngeal dysaesthesia occurred, subsequent O was administered as a 6-h infusion. If grade 3/4 diarrhoea or stomatitis occurred despite appropriate fluoropyrimidine dose reductions, subsequent O was reduced to 100 mg m^−2^. Capecitabine was stopped if pts developed grade 2–3 stomatitis, diarrhoea or nausea and vomiting. If grade 3 toxicity was controlled adequately within 2 days and on resolution of grade 2 toxicity, X was continued at full dose. If grade 2 toxicity occurred a second time, X was dose reduced by 25%, a third time by 50% and if it occurred a fourth time treatment was discontinued. If grade 3 toxicity took longer than 2 days to resolve, X was dose reduced by 25%. If grade 3 toxicity occurred a second time X was dose reduced by 50% and discontinued if it occurred a third time. If grade 4 stomatitis, diarrhoea or nausea and vomiting occurred, X could either be discontinued or dose reduced by 50%, at the investigator's discretion. For X associated PPE, pyridoxine 50 mg tds was commenced and the following modifications were made: grade 1 – no modification, grade 2 – X stopped until resolution and then dose reduced by 15%, grade 3 – X stopped until resolution and then dose reduced by 30%, for recurrent grade 3 – X stopped until resolution and then dose reduced by 50%.

### Assessment of response

Response is assessed according to the revised WHO criteria with RECIST guidelines (Therasse *et al*, 2000). A complete response (CR) is the disappearance of all target lesions. A partial response (PR) is at least a 30% decrease in the sum of the longest diameter of target lesions, taking as reference the baseline sum longest diameter. Progressive disease is at least a 20% increase in the sum of the longest diameter of a target lesion, taking as reference the smallest sum longest diameter recorded since the treatment started or the appearance of one or more new lesions. Stable disease is neither sufficient shrinkage for a partial response, nor sufficient increase to qualify for progressive disease, taking as reference the smallest sum longest diameter since the treatment started. Endoscopic response is used to assess the primary tumour: CR is no evidence of endoluminal disease and negative histology on biopsy; PR in the primary site must have CT evidence of a PR and 50% reduction in the endoscopic appearances of the tumour; SD must be shown on CT scanning; PD at endoscopy classifies overall as PD regardless of response at other evaluable sites.

Patients completed the EORTC QLQ-C30 version 3.0 QOL assessment prior to randomisation and at 3, 6, 9 and 12 months (Aaronson *et al*, 1993).

### Statistical methods

The primary end point of this phase III trial is overall survival and secondary end points are failure-free survival, response, duration of response, time to progression, toxicity and QOL. Patient numbers are based on a 1-year survival of 35% for ECF. A planned total of 1000 pts will be randomised allowing noninferiority to be demonstrated. An improvement in 1-year survival with any of the joint arm combinations (e.g. ECF+EOF *vs* ECX+EOX) from 35 to 44% could be detected with at least 80% power, or for an improvement from 35 to 45% with at least 90% power (two-sided test, alpha 5%). An improvement in 1-year survival with any of the combinations could be detected from 35 to 46% with 90% (two-sided test, alpha=5%). Randomisation is allocated by telephone, and is stratified for performance status, centre and locally advanced *vs* metastatic disease. We are now reporting the two planned interim analyses within this study.

## RESULTS

A total of 204 pts were randomised from 18 oncology centres in the UK between June 2000 and October 2002. The first 80 pts were recruited from four centres and in October 2001, the toxicity data on these pts was analysed and presented to the data monitoring committee (first planned interim analysis). Of the 204 pts, two were ineligible (one had a synchronous lung primary, one was unable to start treatment within 30 days of baseline CT evaluation), three were randomised but did not receive treatment (one patient withdrew consent and two deteriorated clinically and were withdrawn from the study prior to starting treatment) and one patient received treatment in violation with the protocol. A total of 198 pts were, therefore, evaluable for toxicity and response. In total, 53 pts were randomised to ECF, 48 to ECX, 55 to EOF and 48 to EOX. Treatment groups were well balanced for patient characteristics (Table 1).

**Figure 2:**
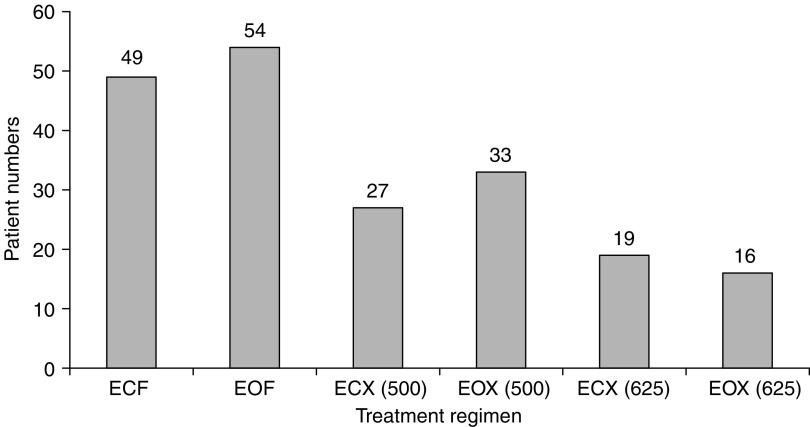
Number of patients and fluoropyrimidine dose. Key: X(500)=capecitabine 500 mg m^−2^ b.i.d.^−1^. X(625)=capecitabine 625 mg m^−2^ b.i.d.^−1^.

In the first planned interim analysis, the first 80 pts were analysed for toxicity. Fluoropyrimidine-related toxicity (stomatitis, palmar-plantar erythema (PPE) and diarrhoea) was analysed according to fluoropyrimidine-containing regimen. The grade 3/4 fluoropyrimidine-related toxicity, in pts receiving X 500 mg m^−2^ b.i.d., was 5.1%; hence, the dose of X was escalated to 625 mg m^−2^ b.i.d. (Tebbutt *et al*, 2002). The trial protocol was amended to include this dose escalation and ethical approval was obtained by all the participating centres. The second interim analysis was performed when 204 pts had been recruited. Figure 2 illustrates the number of pts receiving each of the fluoropyrimidine doses: 103 pts received F 200 mg m^−2^ day^−1^, 60 pts X 500 mg m^−2^ b.i.d.^−1^ and 35 pts X 625 mg m^−2^ b.i.d.^−1^

### Toxicity

Median number of chemotherapy cycles administered was 6 in each of the treatment arms. Grade 3/4 fluoropyrimidine-related toxicity (estimated by patient not cycle) according to regimen and dose of fluoropyrimidine is shown in Table 2. The overall percentage of grade 3/4 fluoropyrimidine-related toxicity in pts receiving 5FU 200 mg m^−2^ day^−1^ was 13.7% (95% CI 7.4–22%), for pts receiving X 500 mg m^−2^ b.i.d. 8.4% (95% CI 2.8–18.7) and for pts receiving X 625 mg m^−2^ b.i.d. 14.7% (95% CI 4.9–31). All other toxicity was predictable and is shown in Table 3, according to treatment arm and in Table 4 according to treatment arm and dose of fluoropyrimidine.

### Dose intensity

There were no significant differences in the dose intensity of fluoropyrimidine, E or platinum agent between the four treatment arms. The dose intensity of the drugs in ECF was E 90.7%, cisplatin 91.9%, 5FU 88.6%; for ECX it was E 90.3%, cisplatin 87.5%, X 87.1%; for EOF it was E 90%, O 89.4%, 5FU 82.1%; for EOX it was E 87.5%, O 87.1%, X 83.3%. Dose intensity according to fluorpyrimidine dosage is shown in Table 5.

### Response

Of the 204 patient's randomised, 198 pts received chemotherapy within the clinical trial. Two pts had nonevaluable disease and therefore 196 pts were included in the response analysis on 12th May 2003. Objective response rates were seen in 15 pts (one CR, 14 PRs) treated with ECF for a response rate of 31%, 21 pts (three CRs, 18 PRs) treated with EOF for a response rate of 39%, 16 pts (four CRs, 12 PRs) treated with ECX for a response rate of 35% and 23 pts (one CR, 22 PRs) treated with EOX for a response rate of 48% (Table 6). The corresponding rates of progressive disease (PD) were 27% with ECF, 20% with EOF, 24% with ECX and 15% with EOX. There were 18 pts (three ECF, seven EOF, five ECX and three EOX) in whom no assessment was available. Of the eight pts who achieved a CR, four were achieved after downstaging chemotherapy and surgery.

The objective response rates of patients receiving fluoropyrimidine or platinum agent are shown Tables 7 and 8, respectively. Objective responses were seen in 36 pts (four CRs, 32 PRs) treated in either of the two 5FU-containing arms (ECF +EOF) for a response rate of 36%, and in 39 pts (five CRs, 34 PRs) treated in either of the two X-containing arms (ECX+EOX) for a response rate of 41%. Objective responses were seen in 31 pts (five CRs, 26 PRs) treated in either of the cisplatin-containing arms (ECF+ECX) for a response rate of 33%, and in 44 pts (four CRs. 40 PRs) treated in either of the O-containing arms (EOF+EOX) for a response rate of 43%.

## DISCUSSION

The purpose of the planned interim analyses presented in this paper was to establish the optimal dose of continuously administered X to be used in combination with either E and cisplatin or E and O in pts with advanced OG cancer, and to confirm the safety of any dose adjustment. The results of the first interim analysis demonstrated grade 3/4 fluoropyrimidine toxicity in 5.1% of pts receiving X 500 mg m^−2^ b.i.d.^−1^ (Tebbutt *et al*, 2002). This resulted in the protocol planned dose escalation of X from 500 to 625 mg m^−2^ b.i.d.^−1^. The second analysis, performed when 204 pts had been randomized, confirmed that the dose of 625 mg m^−2^ b.i.d.^−1^ was safe. In all, 14.7% of pts treated with X 625 mg m^−2^ b.i.d.^−1^ experienced grade 3/4 fluoropyrimidine-related toxicity, which parallels that of PVI 5FU (13.7%), confirming that this is the optimal dose of X to be used in combination with either E and cisplatin or E and O.

A previous phase I study has evaluated the combination of X with E 50 mg m^−2^ and C 60 mg m^−2^, in pts with advanced OG cancer (Evans *et al*, 2002). They evaluated the dose-limiting toxicity (DLT) and maximum tolerated dose of X with escalations of X up to 1250 mg m^−2^ b.i.d.^−1^ D1–14 with 7 days rest. The recommended dose of X for use on an intermittent schedule in combination with E and cisplatin was 1000 mg m^−2^ b.i.d.^−1^. There have been no phase I studies looking at this combination using a continuous schedule of X; however, the results of our study have identified X 625 mg m^−2^ b.i.d.^−1^ to be the optimal dose in both an ECX and EOX regimen based on the similar incidence of fluoropyrimidine-related toxicity (13.7% in 5FU-containing arms and 14.7% in X 625 mg m^−2^ b.i.d.^−1^-containing arms). The non-fluoropyrimidine-related toxicity in all arms of this study was predictable and consistent with previous clinical trials (Ross *et al*, 2002). The rates of grade 3/4 febrile neutropenia were 8% ECF, 14% EOF, 5% ECX and 10% EOX. However, the rate of grade 3/4 febrile neutropenia in pts receiving EOX where X=625 mg m^−2^ b.i.d.^−1^ was 19% (three out of 16 pts), which is higher than any of the other arms. This may be due to relatively small patient numbers; however, the final analysis of this study will establish whether this is significant.

The primary end point of this study is to compare the overall survival in the four treatment arms and thus no survival data will be analysed until accrual is complete. The response rates indicate that X has efficacy similar to 5-FU and that O appears to have promising activity in advanced OG cancer. It was not in the remit of this analysis to compare response rates and to draw firm conclusions from them at this stage would be erroneous. Patient preference is for oral chemotherapy regimen (Borner *et al*, 2002) and with the associated complications of the indwelling central venous catheter necessary with PVI-5FU, the replacement of 5FU with X would clearly be one of considerable benefit to pts.

In summary, we have presented the results of two planned interim analyses within a multicentre randomised study evaluating the role of X and O in advanced OG cancer. The results clearly confirm the safety of the dose escalation of X and demonstrate X 625 mg m^−2^ b.i.d.^−1^ continuously to be the dose that should be used in combination with the EC and EO regimens. In addition, our results show that X has similar efficacy to PVI 5-FU. The trial will continue to accrue to a planned target of 1000 pts.

## Figures and Tables

**Table 1:** Patient characteristics

	**No. of patients**			
**Treatment arm**	**ECF**	**EOF**	**ECX**	**EOX**
Randomisation (*n*)	53	55	48	48
*Age (years)*				
Median	64	61	62	64
Range	40–77	45–76	34–81	37–79
				
*Sex*				
Female	9	8	14	12
Male	39	40	41	41
				
*Performance status*				
0/1	46	48	42	42
2	7	7	6	6
				
*Primary site*				
Oesophagus	21	20	13	14
OGJ	19	15	16	15
Gastric	12	20	18	19
Unknown	1	0	1	0
				
*Histology*				
Adenocarcinoma	44	48	41	41
Squamous carcinoma	5	6	6	5
Other	3	1	0	1
Unknown	1	0	1	0
				
*Disease status*				
Locally advanced	15	11	12	17
Metastatic	38	44	36	31
				
Ineligible	1	0	1	0
Randomised but not treated	3	0	0	0

ECF=epirubicin, cisplatin, 5-fluorouracil; ECX=epirubicin, cisplatin, capecitabine; EOF=epirubicin, oxaliplatin, 5-fluorouracil; EOX=epirubicin, oxaliplatin, capecitabine; OGJ=oesophagogastric junction.

**Table 2:** Fluoropyrimidine toxicity according to treatment arm and dose of fluoropyrimidine

	**Number of patients (%)**								
**Toxicity (grade 3/4)**	**ECF 200**		**EOF 200**	**ECX 500**		**EOX 500**	**ECX 625**		**EOX 625**
Stomatitis	0 (0)		5 (10)	0 (0)		0 (0)	0 (0)		0 (0)
Palmar-plantar erythema	2 (4)		1 (2)	1 (4)		2 (6)	3 (16)		1 (6)
Diarrhoea	1 (2%)		5 (10)	0 (0)		2 (6)	0 (0)		1 (6)
Any 5-FU-related toxicity		13 (13.7)			5 (8.4)			5 (14.7)	
		95% CI 7.4–22			95% CI 2.8–18.7			95% CI 4.9–31	

ECF=epirubicin, cisplatin, 5-fluorouracil; ECX=epirubicin, cisplatin, capecitabine; EOF=epirubicin, oxaliplatin, 5-fluorouracil; EOX=epirubicin, oxaliplatin, capecitabine.

**Table 3:** Non-fluoropyrimidine toxicity

	**ECF**		**EOF**		**ECX**		**EOX**	
**Toxicity (grade 3/4)**	**No. of pts/total**	**%**	**No. of pts/total**	**%**	**No. of pts/total**	**%**	**No. of pts/total**	**%**
Nausea^a^	2/47	4	7/50	14	1/43	2	4/48	8
Alopecia^b^	21/47	50	18/50	36	17/43	40	13/48	27
Lethargy	8/46	17	10/50	20	4/43	9	8/48	17
Peripheral neuropathy^a^	0/47	0	3/50	6	1/43	2	2/48	4
Neutropenia	16/47	34	12/49	24	16/41	39	19/48	40
Thrombocytopenia^a^	2/47	4	4/49	8	4/41	10	3/48	6
Infection^a^	6/47	13	10/50	20	2/43	5	6/48	12
Febrile neutropenia	2/24	8	4/29	14	1/24	5	3/30	10
Anaemia	8/47	17	2/49	4	3/41	7	4/48	8

a No grade 4 toxicity.

b CTC grade 2 alopecia.

Pts=patients; ECF=epirubicin, cisplatin, 5-fluorouracil; ECX=epirubicin, cisplatin, capecitabine; EOF=epirubicin, oxaliplatin, 5-fluorouracil; EOX=epirubicin, oxaliplatin, capecitabine.

**Table 4:** Non-fluoropyrimidine toxicity according to fluoropyrimidine dose

	**ECF 200**		**EOF 200**		**ECX 500**		**EOX 500**		**ECX 625**		**EOX 625**	
**Toxicity (grade 3/4)**	**No. of pts/total**	**%**	**No. of pts/total**	**%**	**No. of pts/total**	**%**	**No. of pts/total**	**%**	**No. of pts/total**	**%**	**No. of pts/total**	**%**
Nausea^a^	2/47	4	7/50	14	1/27	4	2/32	6	0/16	0	2/16	12
Alopecia^b^	21/47	50	18/50	36	12/27	44	10/32	31	5/16	31	3/16	19
Lethargy	8/46	17	10/50	20	3/27	11	5/32	16	1/16	6	3/16	19
Peripheral neuropathy^a^	0/47	0	3/50	6	1/27	4	0/32	0	0/16	0	2/16	12
Neutropenia	16/47	34	12/49	24	9/25	36	15/32	46	6/15	40	4/16	25
Thrombocytopenia^a^	2/47	4	4/49	8	4/26	15	2/32	6	0/15	0	1/16	6
Infection^a^	6/47	13	10/50	20	1/27	4	2/32	6	1/16	6	4/16	25
Febrile neutropenia	2/24	8	4/29	14	1/10	10	0/14	0	0/14	0	3/16	19
Anaemia	8/47	17	2/49	4	2/26	8	3/32	9	1/15	7	1/16	6

a No grade 4 toxicity.

b CTC grade 2 alopecia.

Pts=patients; ECF=epirubicin, cisplatin, 5-fluorouracil; ECX=epirubicin, cisplatin, capecitabine; EOF=epirubicin, oxaliplatin, 5-fluorouracil; EOX=epirubicin, oxaliplatin, capecitabine.

**Table 5:** Dose intensity of chemotherapy agents received according to fluoropyrimidine and dose

		**% of chemotherapy received**		
	**No. of pts**	**5FU/capecitabine (%)**	**Epirubicin (%)**	**Platinum agent (%)**
5FU 200 mg m^−2^	104	85.2	90.3	90.6
Capecitabine 500 mg m^−2^ b.i.d.^−1^	59	87.2	88.9	87.8
Capecitabine 625 mg m^−2^ b.i.d.^−1^	35	81.8	88.8	86.4

Pts=patients; ECF=epirubicin, cisplatin, 5-fluorouracil (5FU); ECX=epirubicin, cisplatin, capecitabine; EOF=epirubicin, oxaliplatin, 5-fluorouracil; EOX=epirubicin, oxaliplatin, capecitabine.

**Table 6:** Response rates according to treatment arm

	**ECF**		**EOF**		**ECX**		**EOX**	
	**No. of pts**	**%**	**No. of pts**	**%**	**No. of pts**	**%**	**No. of pts**	**%**
CR	1	2	3	6	4	9	1	2
PR	14	29	18	33	12	26	22	46
Overall (CR+PR)	15	31	21	39	16	35	23	48
95% CI		(18.7–46.3)		(25.9–53.1)		(21.4–50.3)		(33.3–62.8)
Stable disease	17	36	16	30	14	30	15	31
PD	13	27	11	20	11	24	7	15
No assessment available	3	6	6	11	5	11	3	6

Pts=patients; ECF=epirubicin, cisplatin, 5-fluorouracil; ECX=epirubicin, cisplatin, capecitabine; EOF=epirubicin, oxaliplatin, 5-fluorouracil; EOX=epirubicin, oxaliplatin, capecitabine; CR=complete response; PR=partial response; PD=progressive disease; 95% CI=95% confidence interval.

**Table 7:** Response rates of patients receiving fluoropyrimidine treatment

	**5-FU arms**		**Capecitabine arms**	
	**No. of pts**	**%**	**No. of pts**	**%**
CR	4	4	5	5
PR	32	32	34	36
Overall	36	36	39	41
95% CI		(26.6–46.2)		(31.4–52.1)
SD	33	32	29	30
PD	24	24	18	19
No assessment available	9	8	8	10

Pts=patients; CR=complete response; PR=partial response; PD=progressive disease; SD=stable disease; 95% CI=95% confidence interval.

**Table 8:** Response rates of patients receiving platinum treatment

	**Cisplatin arms**		**Oxaliplatin arms**	
	**No. of pts**	**%**	**No. of pts**	**%**
CR	5	5	4	4
PR	26	28	40	39
Overall	31	33	44	43
95% CI		(23.6–43.3)		(34.1–54.3)
SD	31	33	31	30
PD	24	26	18	18
No assessment available	8	8	9	9

Pts=patients; CR=complete response; PR=partial response; PD=progressive disease; SD=stable disease; 95% CI=95% confidence interval.
